# Retraction: Evaluation of chemopreventive effects of *Acanthus ilicifolius* against azoxymethane-induced aberrant crypt foci in the rat colon

**DOI:** 10.1371/journal.pone.0332843

**Published:** 2025-09-22

**Authors:** The PLOS One Editors

After this article [1] was published, concerns were raised regarding results presented in Figs 4-6.

Specifically:

- The following panels appear to partially overlap:
  - Fig 4B and Fig 4E
  - Fig 5B in [1] and Fig 1D in [2, retracted in 3]
- Fig 5A in [1] appears similar to Fig 5C in [4, corrected in 5 and retracted in 6]
- Fig 6 appears to show the quantification results of Fig 5.

Co-corresponding author AAA stated that there was an error in figure assembly for Fig 4 and that the original underlying data for Figs 4 and 5B are no longer available. They also stated that Fig 5A in [1] is correct and provided the original underlying data for Fig 5A, but did not provide individual-level underlying data for Fig 6. Co-corresponding author AAA provided replicate data from the time of the original experiments for Figs 4B, 4E, and 5B, however, PLOS have concerns with the integrity of the provided replicate data, and have not been able to obtain verification of these data.

In the absence of the original underlying data and independent verification of the replicate data, the above concerns cannot be resolved. Therefore, the *PLOS One* Editors retract this article.

AAA did not agree with the retraction and stands by the article’s findings. MAA, RSA, AS, SDS, and MAA either did not respond directly or could not be reached.
